# Supplementary material for: Outer Membrane Vesicles From The Gut Microbiome Contribute to Tumor Immunity by Eliciting Cross-Reactive T Cells
Source: Front Oncol. 2022 Jun 30;12:912639. doi: 10.3389/fonc.2022.912639 (PMC9281500; doi:10.3389/fonc.2022.912639)
Supplement: Supplementary file 1 [file DataSheet_1.docx]

Supplementary Material

# Supplementary Figures and Tables

## Supplementary Figures

**Supplementary Figure 1. Construction of *E. coli* strains engineered for Lpp-OVA fusion protein expression.** Schematic representations of the construction of EcN(*lpp-OVA*) (**A**) and *E. coli* BL21(DE3)Δ*ompA*(*lpp-OVA*) (**B**). EcN and BL21(DE3)Δ*ompA* were first transformed with the pCas9λred plasmid expressing the λ-Red proteins (orange circles), the Cas9 endonuclease (yellow rectangle) and the tracrRNA. Subsequently, EcN(pCas9λred) and BL21(DE3)Δ*ompA*(pCas9λred) were co-transformed with pCRISPR-*lpp*-sgRNA or pCRISPR-*lpp*-gRNA respectively and with a synthetic donor DNA (dDNA) carrying the OVA nucleotide sequence (in red) flanked by DNA fragments (in black) complementary to *lpp* (in blue) and the genome (in grey). In the system adopted for EcN(*lpp-OVA*) construction, the Cas9 enzyme is guided by a single RNA molecule (*lpp*-sgRNA) containing both the guide RNA and the Cas9-binding hairpin. Instead, in the system adopted for BL21(DE3)Δ*ompA*(*lpp-OVA*) construction, the Cas9 enzyme is guided by an RNA molecule deriving from the hybridization of the *lpp*-gRNA guide RNA with the tracrRNA coded from pCas9λred. Engineered clones with the *lpp-OVA* fusion gene were cured from plasmids. In the schematized resulting cells (bottom), the sequence of the C-terminal portion of the Lpp-OVA fusion protein is reported.

**Supplementary Figure 2. Expression of heterologous antigens in bacteria cells and OMVs.** (**A**) SDS-PAGE and (**B**) Western Blot of total cell extracts (TE) and OMVs of *E. coli* strains EcN(*lpp-OVA*) and BL21(DE3)Δ*ompA*(*lpp-OVA*) (shortened to Δ*ompA*(*lpp-OVA*)) expressing Lpp-OVA fusion protein. For Western Blot anti-OVA polyclonal antibodies were used. (**C**) SDS-PAGE of OMVs derived from *E. coli* strains BL21(DE3)Δ*ompA*(pET-MBP-AH1) and BL21(DE3)Δ*ompA*(pET-FhuD2-SV40) expressing MBP-AH1 and FhuD2-SV40 fusion proteins, respectively. (**D**) Western Blot of OMVs derived from *E. coli* strain BL21(DE3)Δ*ompA*(pET-FhuD2-SV40) expressing FhuD2-SV40 fusion protein using anti-SV40 polyclonal antibodies. EcN and BL21(DE3)Δ*ompA* (shortened to Δ*ompA*) were used as negative controls. Bands corresponding to recombinant antigens are indicated in SDS-PAGE panels by a red arrow. Protocols for sample preparation are described in supplementary material and methods.

**Supplementary Figure 3. Percentages of CD3^+^, CD4^+^, CD8^+^ and OVA-specific CD8^+^ cells from the *lamina propria*.** Mice were treated by oral administration of EcN (black bars) or EcN(*lpp-OVA*) (white bars) and the composition of the *lamina propria* immune cells was analyzed by flow cytometry. Statistical analysis was performed using Student’s t-test (two-tailed). ** P ≤ 0.01.

**Supplementary Figure 4. Tumor growth in control group mice challenged with OVA-B16F10 cells and treated with EcN, OMVs_EcN_, OMVs_ΔompA_ or PBS.** Comparison of the effect of the different control treatments with PBS (placebo).

**Supplementary Figure 5. Percentages of CD3^+^, CD4^+^, CD8^+^ and OVA-specific CD8^+^ cells from tumor infiltrating lymphocytes (TILs).** Mice were treated by oral administration of (**A**) EcN or EcN(*lpp-OVA*), (**B**) OMVs_EcN_ or Lpp-OVA-OMVs_EcN_, and (**C**) OMVs_ΔompA_ or Lpp-OVA-OMVs_ΔompA_ and challenged with OVA-B16F10 cells. At the end of the experiment, tumors were collected and the composition of infiltrating immune cells was analyzed by flow cytometry. Black bars represent the wild type condition whereas white bars represent genetically modified bacteria or OMVs carrying the Lpp-OVA antigen. Statistical analysis was performed using Student’s t-test (two-tailed). * P ≤ 0.05; ** P ≤ 0.01.

**Supplementary Figure 6. Characterization of the TCR β chain repertoire**. (**A**) Normalized Shannon-Wiener index highlights the reduced diversity observed in the immune repertoire of CD8^+^ T cells infiltrating the tumor of EcN(*lpp-OVA*)-treated mice compared to the EcN group. (**B**) Heat map showing the identification of CDR3 similarity motifs using the GLIPH2 algorithm. Fisher exact test is performed to estimate the bias of the pattern identified in the experimental datasets compared to a reference dataset. The Table shows the S%DNSGNT specificity group and the corresponding CDR3 amino acid sequences. The % indicates positions in the CDR3 sequences allowing variants. Amino acids in lower case are encoded by codon overlapping with N nucleotides added during the VDJ rearrangement process. TRBV: T cell receptor variable gene of the beta chain; TRBJ: T cell receptor joining gene of the beta chain. TCR sequencing results originated from the *lamina propria* of 3 EcN-treated mice, 3 EcN(*lpp-OVA*)-treated mice, and from the tumor of 5 EcN-treated mice and 2 EcN(*lpp-OVA*)-treated mice. Data derive from one single experiment (see Figure 3).

**Supplementary Figure 7. Flow cytometry analysis of T cells from spleen and PBMCs.** Animals were given OMVs engineered with Lpp-OVA from *E. coli* BL21(DE3)Δ*ompA* as schematized in Figure 1B and cells were isolated from spleens (A) and peripheral blood (B). The frequency of CD8^+^ IFNγ+ T cells was measured after stimulation with OVA-specific peptide. As negative control, an unrelated peptide was used (ctrl).

**Supplementary Figure 8. Relative Abundance of *Escherichia coli* in fecal samples.** Plot representing the percentage of relative abundance of *E. coli* at T_0_, T_1_ and T_2_ in each group treated as depicted in Figure 5A, calculated using MetaPhlAn v.4.0. Data from a single experiment (3-5 mice per group) (see Figure 5).


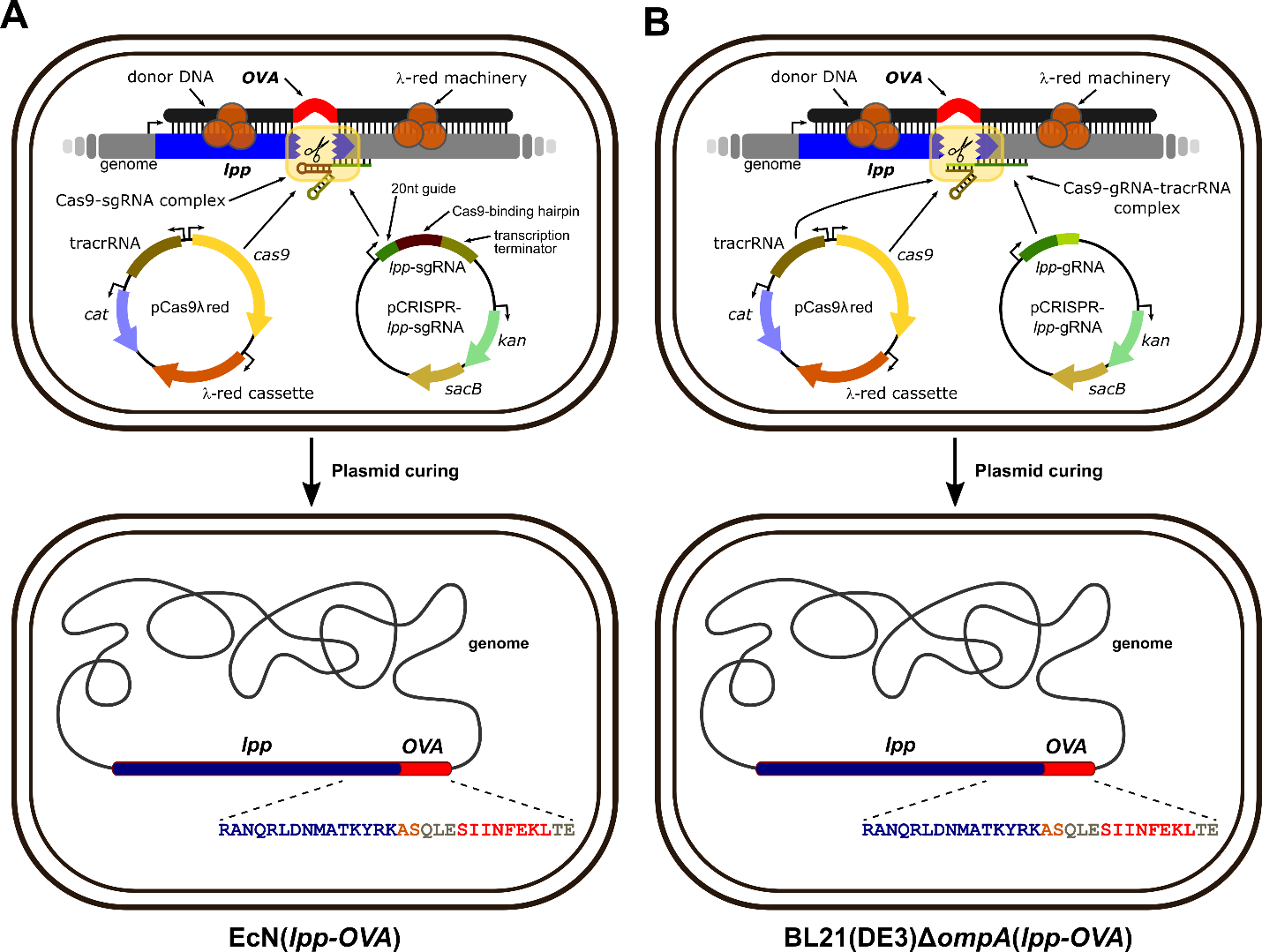


**Supplementary Figure 1.**

**
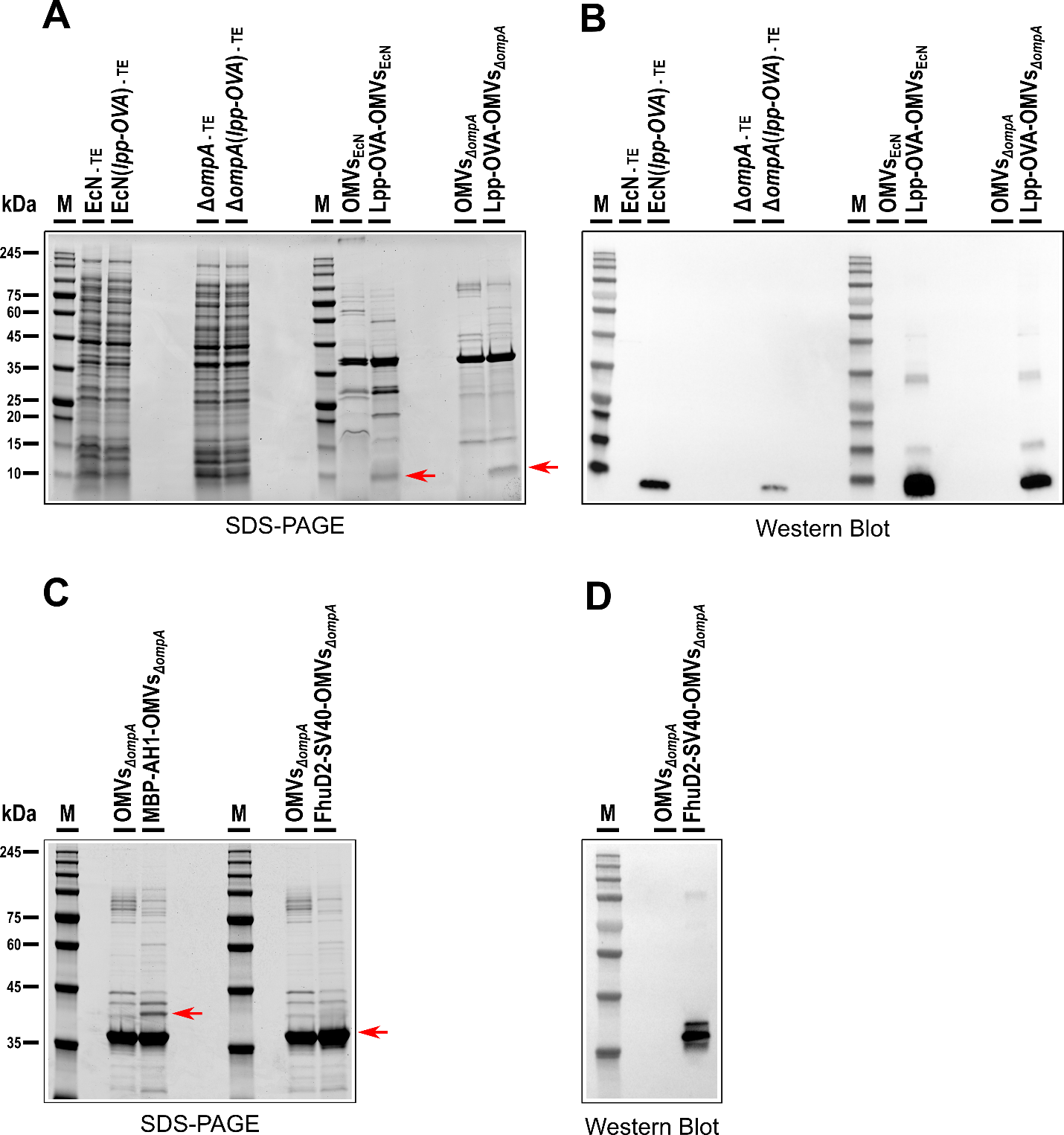
**

**Supplementary Figure 2.**

**
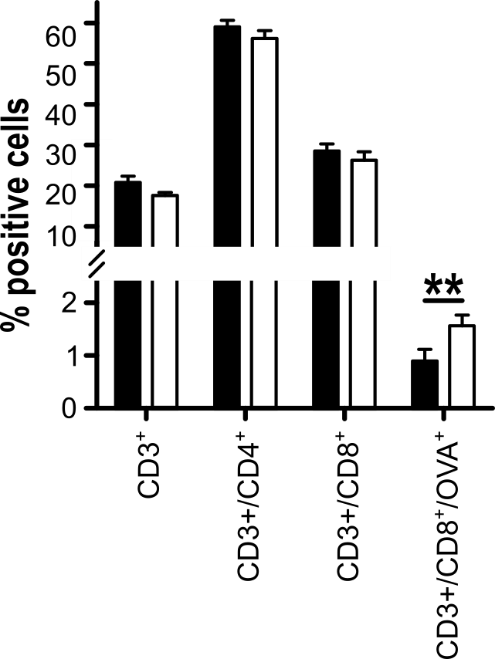
**

**Supplementary Figure 3.**

**
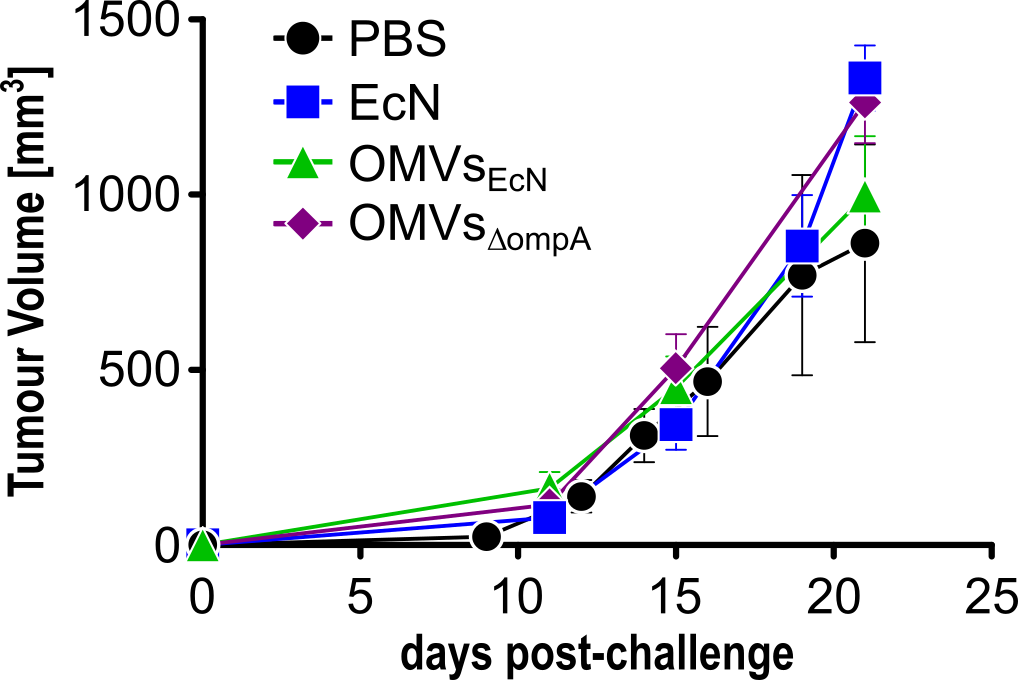
**

**Supplementary Figure 4.**

**
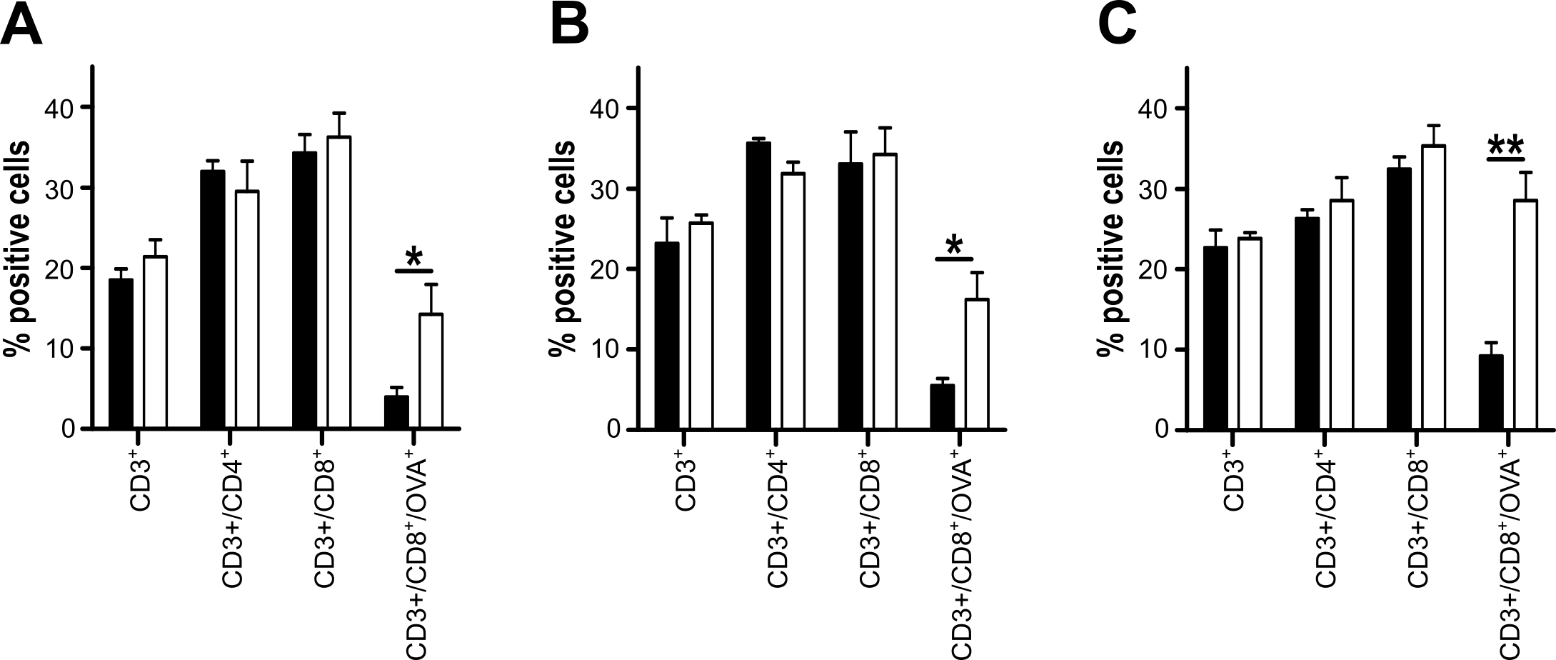
**

**Supplementary Figure 5.**


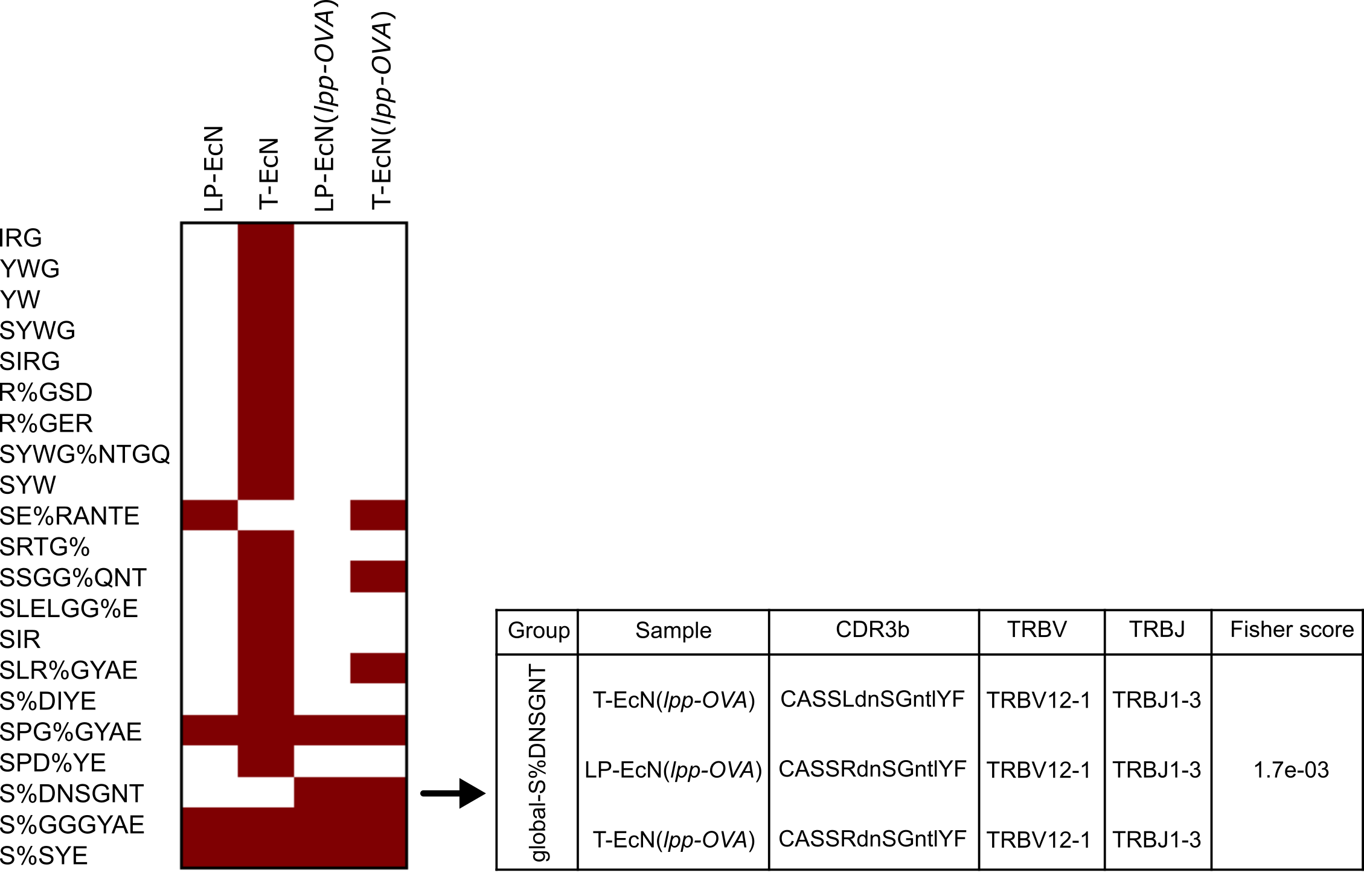


**Supplementary Figure 6.**


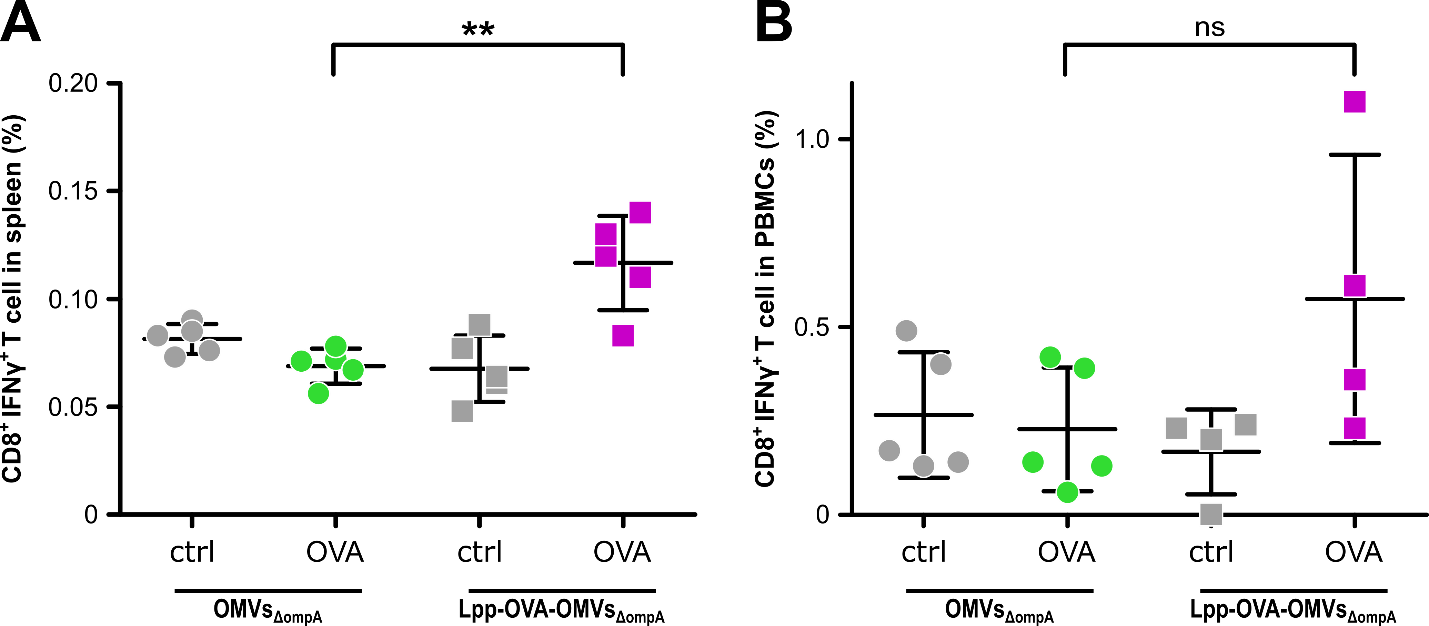


**Supplementary Figure 7.**


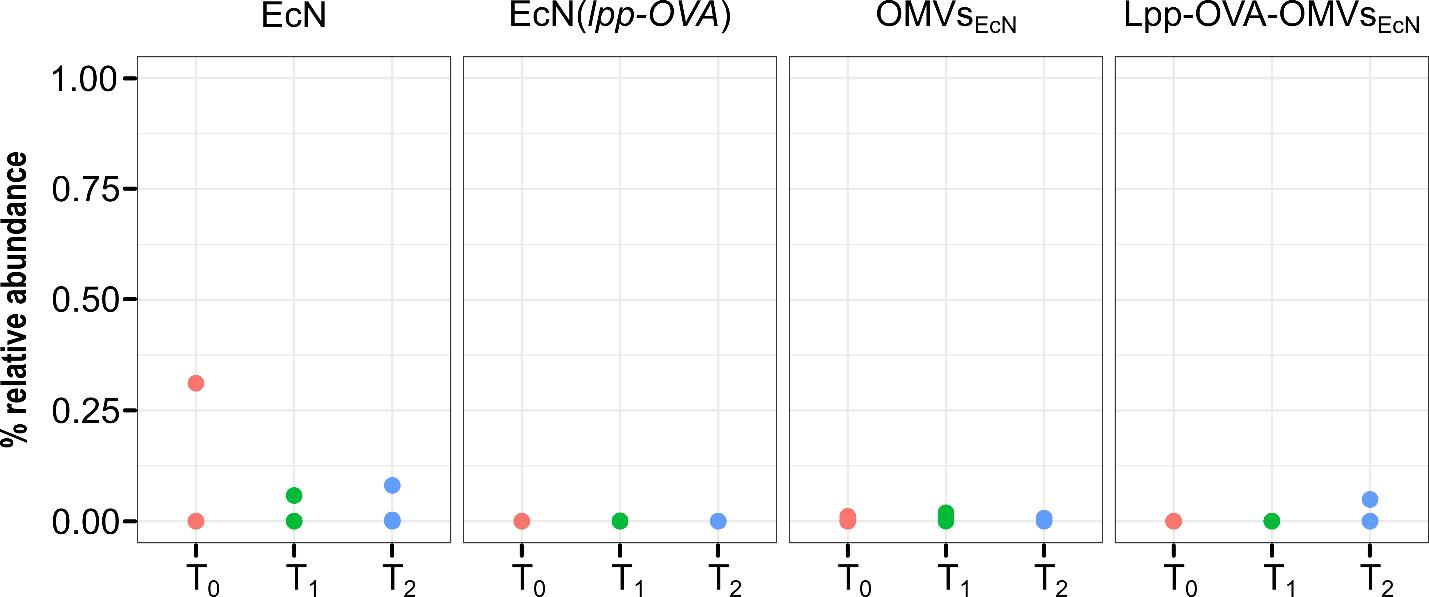


**Supplementary Figure 8.**

Supplementary Table 1. Sequences of guide and donor constructs used for mutagenesis.

| **Name** | **Nucleotide sequence** |
| --- | --- |
| ***lpp*-sgRNA^a^** | CATATTCGTCTCCCTAGGTCTCAAATAAAACGAAAGGCTCAGTCGAAAGACTGGGCCTTTCGTTTTATCTGTTGTTTGTCGGTGAACGCTCTCCTGAGTAGGACAAATACGCATCTGTGCGGTATTTCACACCGGATAAGCTGGATCCTTGACAGCTAGGTCAGTCCTAGGTATAATACTAGT**CGGCATTTCACAGCATTAC**TGTTTTAGAGCTAGAAATAGCAAGTTAAAATAAGGCTAGTCCGTTATCAACTTGAAAAAGTGGCACCGAGTCGGTGCTTTTTTTGAATTCTCTAGAGTCGACCTGCAGAAGCTTAGATCTATTACCCTGTTATCCCTACTCGAGTTC |
| ***lpp*-gRNA^a^** | **CATTTTTCACTTCACAGGTACTATTACTTG** |
| **dDNA^b^** | GGGACTCGAGAAAGCTACTAAACTGGTACTGGGCGCGGTAATCCTGGGTTCTACTCTGCTGGCAGGTTGCTCCAGCAACGCTAAAATCGATCAGCTGTCTTCTGACGTTCAGACTCTGAACGCTAAAGTTGACCAGCTGAGCAACGACGTGAACGCAATGCGTTCCGACGTTCAGGCTGCTAAAGATGACGCAGCTCGTGCTAACCAGCGTCTGGACAACATGGCTACTAAATATCGCAAGGCTAGC**CAGCTGGAAAGCATTATTAACTTTGAAAAACTGACCGAA**TAATGCGGCCGCCATATGTAGTACCTGTGAAGTGAAAAATGGCGCACATTGTGCGCCATTTTTTTGCCTGCTATTTACCGCTACTGCGTCGCGCGTAACATATTCCCTTGCTCTGGTTCCCCATTCTGCGCTGACTCTACTGAAGGCGCATTGCTGGCTGCGGGAGTTGCTCCACTGCTCACCGCAACCGGATACCCTGCCCGACGATACAACGCTTTATCGACTAACTTCTGATCTACAGCCTTATTGTCTTTAAATTGCGTAAAGCCTGCTGGCAGCGTGTACGGCATTGTCTGAACGTTCTGCTGTTCTTCTGCCGATAGTGGTCGATGTACTTCAACATAACGCATCCCGTTAGGTTCCACGGAATATTTCACCGGTTCGTTGATCACTTTCACCGGTGTTCCCGTCCGCAATGCATTGG |

*lpp* guides^a^ and OVA sequence^b^ are in bold.

**Supplementary Table 2. Oligonucleotides used in this study.**

| **Name** | **Nucleotide sequence** |
| --- | --- |
| MB1360 | AAACCATTTTTCACTTCACAGGTACTATTACTTGG |
| MB1361 | AAAACCAAGTAATAGTACCTGTGAAGTGAAAAATG |
| MB1346 | CATATGATGCATCCCGGGACGCGTGAAGACGAAAGGGCCTC |
| MB-1347 | ACGCGTCCCGGGATGCATCATATGACCTCGAGTCCCTATCAG |
| MB1336 | GATGAATCCGATGGAAGCATC |
| *lpp*2 | TCAGTAGAGTCAGCGCAG |
| *lpp*1 | GCTGTCTTCTGACGTTCAGAC |
| MB1337 | GATAAAGCGTTGTATCGTCGG |
| MB1390 | CTAGGACGTTACGCACTGC |
| FhuD2-v-R | TTTTGCAGCTTTAATTAATTTTTC |
| pET-v-F | CATCACCATCACCATCACGATTACA |
| FhuD2-SV40-F | TAATTAAAGCTGCAAAAGGCGGTGATAGCGTGGTG |
| FhuD2-SV40-R | GTGATGGTGATGTTATTACACCATCAGTTTCAGA |

**Supplementary Table 3. Sequences of synthetic DNA encoding Gp70-AH1 and SV40 epitopes.**

| **Name** | **Nucleotide sequence** | **Amino acid sequence** |
| --- | --- | --- |
| **Gp70-AH1 epitope** | ATTCAGGATCCAGCCCAAGCTATGTGTATCACCAATTCGGTTCCTCTCCATCCTATGTTTACCACCAGTTCGGCTCCTCGCCGAGCTATGTTTACCACCAGTTCTGACTCGAGTGAAT | SPSYVYHQFGSSPSYVYHQFGSSPSYVYHQF |
| **SV40 epitope** | GGCGGTGATAGCGTGGTGTATGATTTTCTGAAACTGATGGTG | GGDSVVYDFLKLMV |

**Supplementary Table 4:** CD3^+^, CD4^+^, CD8^+^ and OVA-specific CD8^+^ cell counts from *lamina propria* of mice treated by oral administration of EcN or EcN(*lpp-OVA*) (see Figure 1B). ^a^ number of cells per 250,000 single live cells; ^b^ number of cells per 25,000 CD3^+^ cells; ^c^ number of cells per 5,000 CD8^+^ cells.

| **EcN treated mice** | **CD3^+^ count ^a^ *(%)*** | **CD4^+^ count ^b^ *(%)*** | **CD8^+^ count ^b^ *(%)*** | **OVA^+^ count ^c^ *(%)*** |
| --- | --- | --- | --- | --- |
| **#01** | 42299 *(16.9%)* | 14741 *(59.0%)* | 7048 *(28.2%)* | 43 *(0.62%)* |
| **#02** | 32792 *(19.5%)* | 13812 *(55.2%)* | 7718 *(30.9%)* | 71 *(0.91%)* |
| **#03** | 59601 *(23.8%)* | 14718 *(58.9%)* | 7823 *(31.3%)* | 89 *(1.14%)* |
| **#04** | 56892 *(22.8%)* | 15758 *(63.0%)* | 5897 *(23.6%)* | 59 *(1.01%)* |
| **Average** | **47896 *(20.75%)*** | **14757 *(59.03%)*** | **7121 *(28.50%)*** | **65 *(0.92%)*** |

| **EcN(*lpp-OVA*) treated mice** | **CD3^+^ count ^a^ *(%)*** | **CD4^+^ count ^b^ *(%)*** | **CD8^+^ count ^b^ *(%)*** | **OVA^+^ count ^c^ *(%)*** |
| --- | --- | --- | --- | --- |
| **#05** | 48665 *(19.5%)* | 15032 *(60.1%)* | 5865 *(23.5%)* | 85 *(1.46%)* |
| **#06** | 43773 *(17.5%)* | 14043 *(56.2%)* | 6499 *(26.0%)* | 105 *(1.62%)* |
| **#07** | 43165 *(17.3%)* | 14338 *(57.4%)* | 5806 *(23.2%)* | 107 *(1.86%)* |
| **#08** | 40867 *(16.3%)* | 12702 *(50.8%)* | 8080 *(32.3%)* | 113 *(1.41%)* |
| **Average** | **44118 *(17.65%)*** | **14029 *(56.13%)*** | **6562 *(26.25%)*** | **103 *(1.59%)*** |

**Supplementary Table 5:** CD3^+^, CD4^+^, CD8^+^ and OVA-specific CD8^+^ cell counts from tumor infiltrating lymphocytes of mice treated by oral administration of EcN or EcN(*lpp-OVA*) and challenged with OVA-B16F10 cells (see Figure 1D). ^a^ number of cells per 100,000 single live cells; ^b^ number of cells per 20,000 CD3^+^ cells; ^c^ number of cells per 5,000 CD8^+^ cells.

| **EcN treated mice** | **CD3^+^ count ^a^ *(%)*** | **CD4^+^ count ^b^ *(%)*** | **CD8^+^ count ^b^ *(%)*** | **OVA^+^ count ^c^ *(%)*** |
| --- | --- | --- | --- | --- |
| **#11** | 19643 *(16.3%)* | 6528 *(32.3%)* | 6689 *(38.4%)* | 171 *(3.44%)* |
| **#12** | 21258 *(21.7%)* | 6381 *(33.4%)* | 4866 *(28.8%)* | 132 *(3.87%)* |
| **#13** | 16138 *(16.1%)* | 5629 *(28.1%)* | 6433 *(32.2%)* | 75 *(1.51%)* |
| **#14** | 19756 *(19.8%)* | 6825 *(34.1%)* | 7516 *(37.6%)* | 351 *(7.03%)* |
| **Average** | **19198.8 *(18.5%)*** | **6340.8 *(32.0%)*** | **6376 *(34.3%)*** | **182.3 *(3.97%)*** |

| **EcN(*lpp-OVA*) treated mice** | **CD3^+^ count ^a^ *(%)*** | **CD4^+^ count ^b^ *(%)*** | **CD8^+^ count ^b^ *(%)*** | **OVA^+^ count ^c^ *(%)*** |
| --- | --- | --- | --- | --- |
| **#15** | 17504 *(19.6%)* | 6508 *(32.6%)* | 7754 *(33.4%)* | 622 *(12.4%)* |
| **#16** | 16359 *(16.4%)* | 7764 *(38.8%)* | 6007 *(30.0%)* | 260 *(5.20%)* |
| **#17** | 24095 *(24.1%)* | 4622 *(23.1%)* | 7531 *(37.7%)* | 1180 *(23.06%)* |
| **#18** | 25495 *(25.5%)* | 4708 *(23.5%)* | 8784 *(43.9%)* | 810 *(16.2%)* |
| **Average** | **20863.3 *(21.4%)*** | **5900.5 *(29.5%)*** | **7519.0 *(36.3%)*** | **718.0 *(14.2%)*** |

**Supplementary Table 6:** CD3^+^, CD4^+^, CD8^+^ and OVA-specific CD8^+^ cell counts from tumor infiltrating lymphocytes of mice treated by oral administration of OMVs_EcN_ or Lpp-OVA-OMVs_EcN_ and challenged with OVA-B16F10 cells (see Figure 2G). ^a^ number of cells per 100,000 single live cells; ^b^ number of cells per 20,000 CD3^+^ cells; ^c^ number of cells per 5,000 CD8^+^ cells.

| **OMVs_EcN_ treated mice** | **CD3^+^ count ^a^ *(%)*** | **CD4^+^ count ^b^ *(%)*** | **CD8^+^ count ^b^ *(%)*** | **OVA^+^ count ^c^ *(%)*** |
| --- | --- | --- | --- | --- |
| **#21** | 23528 *(23.3%)* | 6820 *(34.4%)* | 4777 *(26.6%)* | 281 *(3.85%)* |
| **#22** | 17616 *(17.6%)* | 7304 *(36.5%)* | 8061 *(40.3%)* | 192 *(5.62%)* |
| **#23** | 28619 *(28.6%)* | 8375 *(35.9%)* | 6456 *(32.3%)* | 348 *(6.97%)* |
| **Average** | **23254.3 *(23.2%)*** | **7499.7 *(32.3%)*** | **6431.3 *(33.1%)*** | **273.7  *(5.48%)*** |

| **Lpp-OVA-OMVs_EcN_ treated mice** | **CD3^+^ count ^a^ *(%)*** | **CD4^+^ count ^b^ *(%)*** | **CD8^+^ count ^b^ *(%)*** | **OVA^+^ count ^c^ *(%)*** |
| --- | --- | --- | --- | --- |
| **#24** | 23492 *(23.8%)* | 6349 *(33.4%)* | 6381 *(31.4%)* | 660 *(13.2%)* |
| **#25** | 25709 *(26.1%)* | 6579 *(33.1%)* | 6271 *(30.5%)* | 1145 *(22.9%)* |
| **#26** | 27163 *(27.2%)* | 4416 *(29.1%)* | 8168 *(40.8%)* | 620 *(12.4%)* |
| **Average** | **25454.7 *(25.7%)*** | **5781.3 *(31.9%)*** | **6940.0 *(34.2%)*** | **808.3  *(16.2%)*** |

**Supplementary Table 7:** CD3^+^, CD4^+^, CD8^+^ and OVA-specific CD8^+^ cell counts from tumor infiltrating lymphocytes of mice treated by oral administration of OMVs_ΔompA_ or Lpp-OVA-OMVs_ΔompA_ and challenged with OVA-B16F10 cells (see Figure 2G). ^a^ number of cells per 100,000 single live cells; ^b^ number of cells per 20,000 CD3^+^ cells; ^c^ number of cells per 5,000 CD8^+^ cells.

| **OMVs_ΔompA_ treated mice** | **CD3^+^ count ^a^ *(%)*** | **CD4^+^ count ^b^ *(%)*** | **CD8^+^ count ^b^ *(%)*** | **OVA^+^ count ^c^ *(%)*** |
| --- | --- | --- | --- | --- |
| **#31** | 18945 *(18.9%)* | 5136 *(25.7%)* | 5919 *(29.6%)* | 295 *(5.88%)* |
| **#32** | 27846 *(26.5%)* | 5764 *(28.4%)* | 6657 *(32.9%)* | 560 *(11.2%)* |
| **#33** | 22586 *(22.6%)* | 4953 *(24.8%)* | 6968 *(34.8%)* | 525 *(10.5%)* |
| **Average** | **23125.7 *(22.7%)*** | **5284.3 *(26.3%)*** | **6514.7 *(32.4%)*** | **460.0 *(9.19%)*** |

| **Lpp-OVA-OMVs_ΔompA_ treated mice** | **CD3^+^ count ^a^ *(%)*** | **CD4^+^ count ^b^ *(%)*** | **CD8^+^ count ^b^ *(%)*** | **OVA^+^ count ^c^ *(%)*** |
| --- | --- | --- | --- | --- |
| **#34** | 23522 *(23.5%)* | 6761 *(33.8%)* | 6050 *(30.2%)* | 1090 *(21.8%)* |
| **#35** | 26973 *(25.2%)* | 4733 *(24.0%)* | 8273 *(38.0%)* | 1685 *(33.7%)* |
| **#36** | 22713 *(22.8%)* | 5513 *(27.7%)* | 7844 *(37.7%)* | 1500 *(30.0%)* |
| **Average** | **24402.7 *(23.8%)*** | **5669.0 *(28.5%)*** | **7389.0 *(35.3%)*** | **1425.0 *(28.5%)*** |
